# Chemotherapy and prognosis in advanced thymic carcinoma patients

**DOI:** 10.6061/clinics/2015(12)03

**Published:** 2015-12

**Authors:** Zhengbo Song, Xinmin Yu, Yiping Zhang

**Affiliations:** Zhejiang Cancer Hospital, Department of Medical Oncology, Hangzhou, China.

**Keywords:** Thymic carcinoma, Chemotherapy, Prognosis

## Abstract

**OBJECTIVE:**

The role of chemotherapy in treating advanced thymic carcinoma is unclear. The purpose of the current study was to investigate the efficacy of chemotherapy and the prognostic factors for patients with advanced thymic carcinoma.

**METHODS:**

A retrospective review of the medical records of 86 patients treated with chemotherapy for advanced thymic carcinoma was conducted between 2000 and 2012 at our institution. The clinical characteristics, chemotherapy regimens and prognostic factors were analyzed. Survival curves were plotted using the Kaplan–Meier method and the Cox proportional hazard model was used for multivariate analysis.

**RESULTS:**

Of the 86 patients, 56 were male and 30 were female. The median survival time was 24.5 months. For the first-line chemotherapy treatment, the objective response rate was 47.7% and the disease control rate was 80.2%. The median progression-free survival for all patients was 6.5 months for first-line chemotherapy. No significant differences in progression-free survival were observed among the different chemotherapy regimens. Multivariate analyses revealed that the prognostic factors for overall survival included performance status (*p*=0.043), histology grade (*p*=0.048), and liver metastasis (*p*=0.047).

**CONCLUSION:**

Our results suggest that there is no difference in efficacy between multiagent and doublet regimens. The prognosis of patients with advanced thymic carcinoma can be predicted based on histological grade, liver metastasis and performance status.

## INTRODUCTION

Thymic epithelial tumors are the most common anterior mediastinal neoplasm 1. According to worldwide cancer statistics, these tumors are relatively uncommon, with a low incidence of approximately 1.3/1,000,000 people 2. According to the histological criteria published by the World Health Organization (WHO) in 2004, they are classified into two histopathological types: thymoma and thymic carcinomas.

Thymic carcinomas are rare, invasive thymic epithelial tumors associated with a poor prognosis. Surgery is the main therapeutic modality for early-stage patients 3. For people with advanced-stage thymic carcinoma, complete surgical resection is typically not possible, and the only treatment option is palliative chemotherapy or radiochemotherapy 4. Despite the 40–60% response rate to first-line chemotherapy of patients with advanced thymic carcinoma, most of these patients relapse within one year of initial treatment and the majority eventually die due to disease progression.

Because of the rarity of this cancer, published findings regarding the efficacy and toxicity of chemotherapy are limited. Knowledge regarding chemotherapy for thymic carcinoma has mainly been based on retrospective series with small patient samples, although several prospective trials have also been conducted 4. Several studies have described palliative chemotherapy regimens, but because no randomized controlled studies have been performed, it is unclear which regimen is the most effective.

Our current study reviewed a series of consecutive patients with advanced thymic carcinoma who had been treated at a single institution to evaluate the efficacies of various regimens. The prognostic factors for advanced thymic carcinoma were also determined.

## MATERIALS AND METHODS

### Patient eligibility

Patients with pathological stage IV (including IVa and IVb) thymic carcinoma who had undergone treatment at Zhejiang Cancer Hospital between January 2000 and December 2012 were retrospectively identified. The study was approved by the Ethics Committee of Zhejiang Cancer Hospital.

Two pathologists who were blinded to the patients’ clinical and pathological data reviewed all of the samples. The histologic types were determined based on the 2004 WHO classification 5. Patients’ clinical stages were determined according to the Masaoka-Koga staging system 6. Histological findings were classified as low-grade (squamous cell, mucoepidermoid, and basaloid carcinomas) or high-grade (lymphoepithelioma-like, neuroendocrine, clear cell, sarcomatoid and undifferentiated carcinomas) according to Suster and Rosai 7. Recurrence or metastasis was confirmed by chest CT, bone scan and abdomen CT. Patients who died from another disease not related to thymic carcinoma were excluded from the current study.

### Response evaluation and statistical analysis

The Response Evaluation Criteria in Solid Tumors (RECIST 1.1) system was used to evaluate tumor responses. Objective tumor responses included complete response (CR), partial response (PR), stable disease (SD) and progressive disease (PD). The disease control rate (DCR) was defined as the sum of the objective responses and stabilization rates (CR+PR+SD).

Progression-free survival (PFS) encompassed the time from the first cycle of chemotherapy to the time of documented progression or death. Survival was recorded from the first day of treatment to the date of death or that of the last follow-up visit. Survival curves were calculated using the Kaplan–Meier method. Multivariate analysis was performed using the Cox regression model. Statistical analysis was performed with SPSS 17 software (Inc., Chicago, IL). A *p*<0.05 was considered significant. The last follow-up time point was Jan 31, 2014.

## RESULTS

### Patient characteristics

A total of 86 patients who met our criteria were identified, and the patient characteristics are listed in Table 1. The study population consisted of 56 men and 30 women, with a median age at diagnosis of 48 years (range of 31-79). At enrollment, 47 patients (54.7%) had stage IVa disease and 39 (43.3%) had stage IVb disease. Histologic examination revealed the following subtypes of thymic carcinoma: 52 patients (60.5%) had squamous cell carcinoma, 13 (15.1%) had undifferentiated carcinoma, 7 (8.1%) had neuroendocrine carcinoma, 4 (4.7%) had small cell carcinoma, 3 (3.5%) had mucoepidermoid carcinoma and 7 (8.1%) had carcinoma of another subtype. Among the 86 patients, an Eastern Cooperative Oncology Group performance status (PS) of 0–1 was observed in 74 patients (86.0%) and PS 2 (14.0%) was observed in 12 patients.

At the time of treatment, the most common sites of metastasis included the lymph nodes (22 patients, 25.6%), lung (20 patients, 23.3%), liver (14 patients, 16.3%), bone (12 patients, 14.0%) and others (20.8%).

### Chemotherapy regimens

The first-line chemotherapy regimens used are shown in Table 1. The distribution of these regimens was as follows: 7 patients (8.1%) received ADOC (cisplatin, doxorubicin, vincristine and cyclophosphamide); 17 (19.8%) received CAP (cyclophosphamide, doxorubicin, and cisplatin); 12 (14.0%) received VIP (etoposide, ifosfamide and cisplatin); 43 (50.0%) received carboplatin or cisplatin-based doublet chemotherapy (including paclitaxel, gemcitabine, vinorelbine, cyclophosphamide and docetaxel); and 7 (8.1%) received a single agent. Forty-two patients (48.9%) received second-line or greater chemotherapy. The most common regimen was docetaxel-based chemotherapy, which was administered to 16 patients, followed by paclitaxel-based chemotherapy.

### Efficacy analysis of first-line chemotherapy regimens

The median follow-up period was 31.5 (5.5-125.0) months. For first-line chemotherapy, 41 patients exhibited a PR (47.7%), 28 showed SD (32.6%) and 17 exhibited PD. There were no complete responders. The response rate for the patients treated with ADOC chemotherapy (n=7) was 42.9% and it was 51.7% for those treated with triplet (CAP and VIP) chemotherapy (n=29), 48.8% for those treated with platinum-based doublet chemotherapy (n=43), and 28.6% for those who received single-agent chemotherapy (n=7) (Table 2).

**Figure 1:**
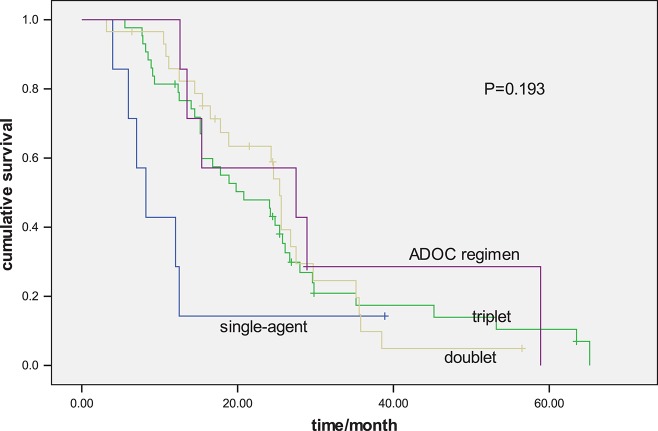
Kaplan–Meier curves comparing PFS in patients treated with various first-line chemotherapy regimens.

**Figure 2:**
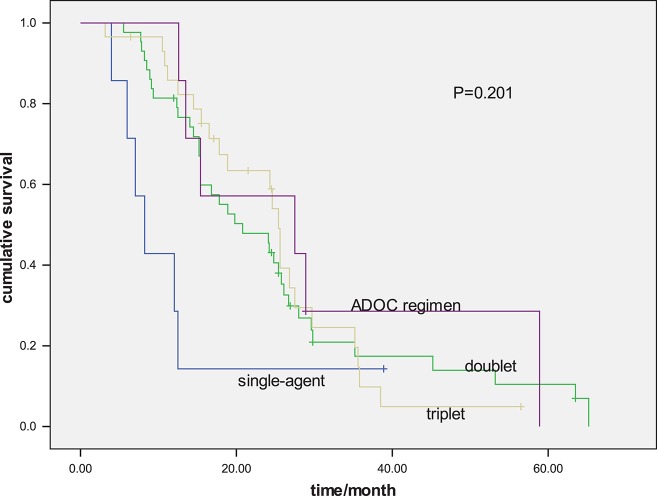
Kaplan–Meier curves comparing OS in patients treated with various first-line chemotherapy regimens.

The median PFS for all patients was 6.5 months. The PFS values for the ADOC regimen and triplet, doublet and single-agent groups were 7.5 months, 6.6 months, 6.6 months and 2.3 months, respectively (*p*=0.193) (Figure 1). The median survival time for all patients was 24.1 months (range of 6.5-125 months). The OS values for the ADOC, triplet, doublet and single-agent groups were 27.5 months, 25.4 months, 20.8 months and 8.2 months, respectively (*p*=0.201) (Figure 2).

### Univariate and Cox regression analyses

**Figure 3:**
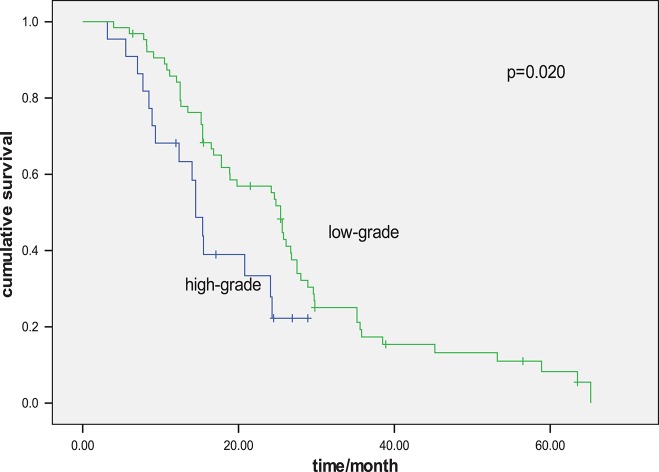
Kaplan–Meier curves comparing OS in patients with different histological grades.

Univariate analyses were performed using the Kaplan–Meier method to assess the predictive capacity of each variable for the determination of OS and the data are summarized in Table 3. Gender, age, cancer stage, chemotherapy regimen and smoking status were not found to be significantly associated with OS. PS (*p*=0.002), histology grade (*p*=0.020), liver metastasis (*p*=0.001, Figure 3) and the response to first-line chemotherapy (*p*=0.047) were predictive of OS.

A multivariate Cox regression model was constructed with the incorporation of PS, histology grade, the response to first-line chemotherapy, cancer stage and liver metastasis to evaluate OS. PS (*p*=0.043), histology grade (*p*=0.048) and liver metastasis (*p*=0.047) remained as independent prognostic factors, but cancer stage (*p*=0.255) and the response to first-line chemotherapy (*p*=0.677) did not have significant influences on survival based on multivariate analysis (Table 4).

## DISCUSSION

To the best of our knowledge, this is the largest study evaluating the efficacy of chemotherapy and prognostic factors for advanced thymic carcinoma. Our results suggest that there are no differences in efficacy among the different regimens used to treat advanced thymic carcinoma. Liver metastasis, poor PS and high-grade histology indicated a worse prognosis.

There is still no consensus on the optimal chemotherapy regimen for advanced thymic carcinoma. Previous studies have shown that multiagent chemotherapy plays an important role in advanced thymic carcinoma 8. Anthracycline-based regimens, such as CAP and ADOC, are widely used and are recommended as standard first-line treatments of advanced thymic carcinoma according to the NCCN guidelines. However, the cardiac toxicity of anthracycline-based regimens is an important consideration in clinical practice 9,10. Non-anthracycline regimens are preferable to minimize potential cardiac toxicity.

The use of platinum-based doublet chemotherapy as first-line chemotherapy has been shown to be efficacious. Regimens such as TC (carboplatin and paclitaxel) and AC (amrubicin and carboplatin) have also been shown to be effective based on studies with small sample sizes 11,15. However, no prospective or retrospective study has compared the efficacies of multiagent and doublet chemotherapy in the treatment of this rare carcinoma. No differences in PFS or OS among the different regimens were observed in the current study; however, the frequency of toxicity was much lower for doublet chemotherapy compared with multiagent chemotherapy (the frequencies of grades 3/4 toxicity were 65.1% and 86.1%, respectively, *p*=0.06).

Few studies have been conducted to identify the prognostic factors for advanced thymic carcinoma. Histologic grade was found to be a significant prognostic factor for overall survival by Hosaka et al. 16, but no statistically significant difference was observed between low- and high-grade histology groups in another study 17. However, in the present study, a significant difference in OS between the low-grade and high-grade histology groups was observed. A retrospective study conducted by Okuma et al. 17, which included 40 advanced thymic carcinoma patients, demonstrated that the response to first-line chemotherapy was the only factor associated with a significantly better prognosis. In contrast, this response was not found to be a significant prognostic factor based on multivariate analysis in the current study. Our results showed that liver metastasis was a poor prognostic factor for advanced thymic carcinoma, consistent with other solid carcinomas 18.

Recent studies have focused on the role of targeted therapy in advanced thymic carcinoma. c-KIT expression has been demonstrated to be common in thymic carcinomas. Several case reports have documented clinical responses to treatment with sunitinib and sorafenib 19-20. Recently, Rajan et al. 21 have shown that cixutumumab, a fully human IgG1 monoclonal antibody that targets insulin-like growth factor 1 receptor and is used as a mono-therapy, is well tolerated and is active in patients with relapsed thymoma but not in those with thymic carcinoma.

The major limitations of the present study are its retrospective nature and the heterogeneity of the chemotherapy regimens evaluated. However, it provides relevant insights into the efficacies of different treatments of advanced thymic carcinoma as one of the largest reports to date focusing on treatment regimens for this rare tumor type.

The results of this study suggest that there is no difference in efficacy between multiagent and doublet regimens (the *p* values for PFS and OS were 0.93 and 0.63, respectively). The prognosis of advanced thymic carcinoma can be predicted based on histological findings, liver metastasis and PS.

## AUTHOR CONTRIBUTIONS

Song Z was responsible for collection of the study data and manuscript writing. Yu X was responsible for manuscript writing. Zhang Y was responsible for collection of the study data and the literature review.

## Figures and Tables

**Table 1:** Demographic characteristics of the study population.Demographic characteristics of the study population.

	Number (%)
Gender	
Male	56(65.1)
Female	30(34.9)
Age	
Range	31-79
Median	48
<50	52(60.5)
≥50	34(39.5)
Smoking status	
Never	32(37.2)
Former/current	54(62.8)
Stage	
IVa	47(54.7)
IVb	39(45.3)
Liver metastasis	
Yes	14(19.4)
No	72(80.6)
PS	
0-1	74(86.0)
2-3	12(14.0)
Histology	
Low-grade	55(64.0)
Squamous cell carcinoma	52(60.0)
Mucoepidermoid carcinoma	3(4.0)
High-grade	31(36)
Undifferentiated carcinoma	13(15.1)
Neuroendocrine carcinomas	7(8.1)
Small cell carcinoma	4(4.7)
Others	7(8.1)
First-line chemotherapy	
ADOC	7(8.1)
Triplet chemotherapies	29(33.7)
Double regimens	43(50.0)
Cisplatin-containing	25(29.1)
Carboplatin-containing	18(20.9)
Single-agent	7(8.1)
Further-line chemotherapy	
Yes	42(48.8)
No	44(51.2)

**ADOC: cisplatin, doxorubicin, vincristine and cyclophosphamide**

**Table 2:** The efficacy of different regimens.

Efficacy	ADOC (n=7)	Triplet (n=29)	Doublet (n=43)	Single-agent (n=7)
Complete response	0	0	0	0
Partial response	3	15	21	2
Stable disease	3	9	13	1
Progressive disease	1	5	9	4
Response rate	42.90%	51.70%	48.80%	28.60%
Disease control rate	85.70%	82.80%	79.10%	42.90%
Median progression-free survival	7.5 months	6.6 months	6.6 months	2.3 months
Median overall survival	27.5 months	25.4 months	20.8 months	8.2 months

**Table 3:** Univariate analysis of the patient survival according to the clinicopathologic characteristics.

	Median PFS	*p*	Median OS	*p*
Gender		0.650		0.722
Male	6.5		24.3	
Female	5.5		24.1	
Age		0.059		0.749
<50	6.6		24.2	
≥50	5.5		18.9	
Smoking status		0.785		0.895
Never	6.6		24.2	
Former/current	6.5		20.8	
Stage		0.126		
IVa	7.5			
IVb	5.5			
PS		0.019		0.002
0-1	6.8		24.8	
2-3	2.3		9.3	
Histology		0.023		0.020
Low-grade	7.5		25.4	
High-grade	4.3		14.5	
**Liver metastasis**		0.013		0.001
**Yes**	2.4		10.8	
**No**	6.8		25.4	
First-line chemotherapy		0.193		0.201
ADOC	7.5		27.5	
Triplet	6.6		25.4	
Double regimens	6.6		20.8	
Single-agent	2.3		8.2	
Further-line chemotherapy		0.725		0.042
Yes	6.4		27.6	
No	6.6		20.8	
Response to first-line chemotherapy		0.014		0.047
Yes	8.2		28.5	
No	4.2		21.5	

**Table 4:** Multivariate survival analysis for disease-Free survival and overall survival.

	PFS			OS		
	HR	95% CI	*p*	HR	95% CI	*p*
Liver metastasis (yes vs. no)	2.716	1.052-50121	0.027	1.757	1.214-3.557	0.047
Histology (Low-grade vs. high-grade)	0.489	0.411-0.978	0.032	0.761	0.475-0.996	0.048
Stage (IVb vs. IVa)	1.24	0.875-3.552	0.127	1.987	0.457-5.213	0.255
PS (2-3/0-1)	2.756	1.542-4.221	0.006	2.712	1.251-5.512	0.043
Response to first-line chemotherapy (yes vs. no)	0.754	0.424-1.572	0.067	0.812	0.517-2.423	0.677

## References

[1] Eng TY, Fuller CD, Jagirdar J, Bains Y, Thomas CR (2004). Thymic carcinoma: state of the art review. Int J Radiat Oncol Biol Phys.

[2] Engels EA, Pfeiffer RM (2003). Malignant thymoma in the United States: Demographic patterns in incidence and associations with subsequent malignancies. Int J Cancer.

[3] Ruffini E, Detterbeck F, Van Raemdonck D, Rocco G, Thomas P, Weder W (2014). Thymic carcinoma: a cohort study of patients from the european society of thoracic surgeons database. J Thorac Oncol.

[4] Wei ML, Kang D, Gu L, Qiu M, Zhengyin L, Mu Y (2013). Chemotherapy for thymic carcinoma and advanced thymoma in adults. Cochrane Database Syst Rev.

[5] Marx A, Strobel PH, Zettl A, Chan JKC, Müller-Hermelink HK, Harris CC (2004). World Health Organization classification of tumours. Pathology and genetics of tumours of the lung, pleura, thymus and heart..

[6] Masaoka A, Monden Y, Nakahara K, Tanioka T (1981). Follow-up study of thymomas with special reference to their clinical stages. Cancer.

[7] Suster S, Rosai J (1991). Thymic carcinoma. A clinicopathologic study of 60 cases. Cancer.

[8] Schmitt J, Loehrer Sr PJ (2010). The role of chemotherapy in advanced thymoma. J Thorac Oncol. J Thorac Oncol..

[9] Fornasiero A, Daniele O, Ghiotto C, Sartori F, Rea F, Piazza M (1990). Chemotherapy of invasive thymoma. J Clin Oncol.

[10] Loehrer PJ, Kim K, Aisner SC, Livingston R, Einhorn LH, Johnson D (1994). Cisplatin plus doxorubicin plus cyclophosphamide in metastatic or recurrent thymoma: final results of an intergroup trial. The Eastern Cooperative Oncology Group, Southwest Oncology Group, and Southeastern Cancer Study Group. J Clin Oncol.

[11] Lemma GL, Lee JW, Aisner SC, Langer CJ, Tester WJ, Johnson DH (2011). Phase II study of carboplatin and paclitaxel in advanced thymoma and thymic carcinoma. J Clin Oncol.

[12] Lemma GL, Lee JW, Aisner SC, Langer CJ, Tester WJ, Johnson DH (2010). Efficacy of chemotherapy with carboplatin and paclitaxel for unresectable thymic carcinoma. Lung Cancer.

[13] Komatsu Y, Koizumi T, Tanabe T, Hatayama O, Yasuo M, Okada M (2006). Salvage chemotherapy with carboplatin and paclitaxel for cisplatin-resistant thymic carcinoma—three cases. Anticancer Res.

[14] Watanabe K, Shinkai M, Goto H, Yoshikawa S, Yamaguchi N, Hara Y (2013). Chemotherapy with carboplatin and paclitaxel after failure of primary chemotherapy for advanced thymic carcinoma. A report of three cases and review of the literature. Tumori.

[15] Inoue A, Sugawara S, Harada M, Kobayashi K, Kozuki T, Kuyama S (2014). Phase II Study of Amrubicin Combined with Carboplatin for Thymic Carcinoma and Invasive Thymoma: North Japan Lung Cancer Group Study 0803. J Thorac Oncol.

[16] Hosaka Y, Tsuchida M, Toyabe S, Umezu H, Eimoto T, Hayashi J (2010). Masaoka Stage and Histologic Grade Predict Prognosis in Patients With Thymic Carcinoma. Ann Thorac Surg.

[17] Okuma Y, Hosomi Y, Takagi Y, Sasaki E, Hishima T, Maeda Y (2013). Clinical outcomes with chemotherapy for advanced thymic carcinoma. Lung Cancer.

[18] Hoang T, Xu R, Schiller JH, Bonomi P, Johnson DH (2005). Clinical model to predict survival in chemonaive patients with advanced non-small-cell lung cancer treated with third-generation chemotherapy regimens based on eastern cooperative oncology group data. J Clin Oncol.

[19] Ströbel P, Bargou R, Wolff A, Spitzer D, Manegold C, Dimitrakopoulou-Strauss A (2010). Sunitinib in metastatic thymic carcinomas: laboratory findings and initial clinical experience. Br J Cancer.

[20] Li XF, Chen Q, Huang WX, Ye YB (2009). Response to sorafenib in cisplatin-resistant thymic carcinoma: a case report. Med Oncol..

[21] Rajan A, Carter CA, Berman A, Cao L, Kelly RJ, Thomas A (2014). Cixutumumab for patients with recurrent or refractory advanced thymic epithelial tumours: a multicentre, open-label, phase 2 trial. Lancet Oncol..

